# Different functional traits among closely related algal symbionts dictate stress endurance for vital Indo‐Pacific reef‐building corals

**DOI:** 10.1111/gcb.15799

**Published:** 2021-08-02

**Authors:** Kenneth D. Hoadley, Daniel. T. Pettay, Allison Lewis, Drew Wham, Chris Grasso, Robin Smith, Dustin W. Kemp, Todd LaJeunesse, Mark E. Warner

**Affiliations:** ^1^ School of Marine Science and Policy University of Delaware Lewes UK; ^2^ Biological Sciences University of Alabama Tuscaloosa Alabama USA; ^3^ Dauphin Island Sea Lab Dauphin Island Alabama USA; ^4^ Department of Biology Pennsylvania State Institutes of Energy and the Environment University Park Pennsylvania USA; ^5^ Science Under Sail Wellington Park QLD Australia; ^6^ Department of Biology University of Alabama Birmingham Alabama USA; ^7^ Present address: University of South Carolina Beaufort South Carolina USA; ^8^ Present address: National Science Foundation Silver Springs Maryland USA; ^9^ Present address: The Nature Conservancy St. Croix US Virgin Islands USA

**Keywords:** *Cladocopium*, comparative physiology, coral bleaching, functional ecology, mutualism, *Porites*, Symbiodiniaceae

## Abstract

Reef‐building corals in the genus *Porites* are one of the most important constituents of Indo‐Pacific reefs. Many species within this genus tolerate abnormally warm water and exhibit high specificity for particular kinds of endosymbiotic dinoflagellates that cope with thermal stress better than those living in other corals. Still, during extreme ocean heating, some *Porites* exhibit differences in their stress tolerance. While corals have different physiological qualities, it remains unknown whether the stability and performance of these mutualisms is influenced by the physiology and genetic relatedness of their symbionts. We investigated two ubiquitous Pacific reef corals, *Porites rus* and *Porites cylindrica*, from warmer inshore and cooler offshore reef systems in Palau. While these corals harbored a similar kind of symbiont in the genus *Cladocopium* (within the ITS2 *C15* subclade), rapidly evolving genetic markers revealed evolutionarily diverged lineages corresponding to each *Porites* species living in each reef habitat. Furthermore, these closely related *Cladocopium* lineages were differentiated by their densities in host tissues, cell volume, chlorophyll concentration, gross photosynthesis, and photoprotective pathways. When assessed using several physiological proxies, these previously undifferentiated symbionts contrasted in their tolerance to thermal stress. Symbionts within *P*. *cylindrica* were relatively unaffected by exposure to 32℃ for 14 days, whereas *P*. *rus* colonies lost substantial numbers of photochemically compromised symbionts. Heating reduced the ability of the offshore symbiont associated with *P*. *rus* to translocate carbon to the coral. By contrast, high temperatures enhanced symbiont carbon assimilation and delivery to the coral skeleton of inshore *P*. *cylindrica*. This study indicates that large physiological differences exist even among closely related symbionts, with significant implications for thermal susceptibility among reef‐building *Porites*.

## Graphical abstract

Written Summary:We investigated two ubiquitous Pacific reef corals, Porites rus and Porites cylindrica, from warmer inshore and cooler offshore reef systems in Palau. While these corals harbored a similar kind of symbiont in the genus Cladocopium, rapidly evolving genetic markers revealed evolutionarily diverged lineages corresponding to each Porites species living in each reef habitat. Physiological differences were observed across these closely related Cladocopium lineages and are shown to play a role in the coral's response to thermal stress. Photos taken by Drs Allison Lewis and Dustin Kemp.

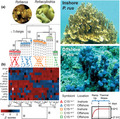

## 1 INTRODUCTION

The evolutionary success and ecological importance of reef‐building corals are underpinned by their mutualistic symbioses with endosymbiotic dinoflagellates in the family Symbiodiniaceae (LaJeunesse et al., 2018; Muscatine & McCloskey, 1981; Trench, 1993). Despite their ecological dominance in the tropics over millions of years while experiencing large shifts in climate, many of these mutualisms are disrupted by small increases in temperature (Hoegh‐Guldberg & Bruno, 2010). The warming of oceans and increasing episodes of high thermal anomalies have led to wide‐scale losses in symbionts (i.e., coral bleaching), mass mortality, and declining coral populations (Hoegh‐Guldberg et al., 2007; Hughes et al., 2019; Pandolfi et al., 2011). Accurately forecasting future coral reef ecosystem success will largely rely on improved understanding of coral physiology and ecology in response to continued ocean warming.

Reef‐forming corals in the genus *Porites* are critical to coral reef ecosystem formation and growth across the Indo‐Pacific and Red Sea regions (Birkeland et al., 2004; DeVantier & Turak, 2017; Monroe et al., 2018) and have contributed to tropical reef building for thousands of years (Kayanne et al., 2002; Montaggioni, 2005). *Porites* are particularly dominant in warm lagoonal and back reef habitats and are thus well adapted for withstanding episodic thermal stressors (Barshis et al., 2018; Berumen et al., 2013; LaJeunesse et al., 2003; Marshall & Baird, 2000; McClanahan, 2004; Monroe et al., 2018). As warming is likely to continue, with further losses in other coral taxa, *Porites* are likely to become increasingly dominant (Carpenter et al., 2008; Loya et al., 2001). The thermal tolerance of most Indo‐Pacific *Porites* is in part attributed to the specific kinds of symbiont that they harbor (Fitt et al., 2009). Nonetheless, variability in bleaching among different *Porites* may relate to species differences, differences among individual genotypes, or possibly unrecognized differences in their symbionts (Boulay et al., 2014; McClanahan, 2004).

The island nation of Palau has diverse coral reef habitats characterized by large environmental gradients (Colin, 2009). In particular, corals from near shore rock island habitats, thrive in warm, low pH, low flow waters, compared to offshore barrier reef habitats (Barkley et al., 2015; Golbuu et al., 2007, 2016; Shamberger et al., 2014; Woesik et al., 2012). Unsurprisingly, during warm‐water anomalies most reef corals in the rock islands show fewer signs of stress (e.g., pigment loss or bleaching) than offshore conspecifics (Bruno et al., 2001; Woesik et al., 2012). While possible reasons for these different stress responses include the animal's acclamatory state (Palumbi et al., 2014) as well as the presence of different locally adapted coral populations (Kenkel & Matz, 2016), dissimilar physiologies of the resident algal symbiont may also contribute to stress tolerance or sensitivity. Many colonies from inshore coral communities harbor symbionts from an evolutionarily divergent genus compared to their counterparts living in offshore habitats, *Durusdinium* vs. *Cladocopium* (Hoadley et al., 2019; LaJeunesse et al., 2004). These differences in host–symbiont pairings contribute to marked differences in how individual coral colonies endure thermal stress (Hoadley et al., 2019). However, corals in the genus Porites, which are critical to reef formation, harbor genetically similar symbionts, *Cladocopium* C15, over broad environmental gradients.

*Cladocopium* is the most globally abundant symbiotic dinoflagellate genus, likely containing hundreds of species distributed broadly across all latitudes and the dominant genus among reef‐building corals in the Pacific (LaJeunesse, 2005; Thornhill et al., 2014; Turnham et al., 2021). While many *Cladocopium* are sensitive to warm temperatures (Hoadley et al., 2019; Hoogenboom et al., 2017), others are exceptionally heat tolerant. Corals harboring *Cladocopium* C15 display minimal photoinactivation and symbiont cell loss when exposed to high temperatures (Fisher et al., 2012; Fitt et al., 2009; Hoadley, Pettay, et al., 2015; Hume et al., 2013). Nearly all *Porites* surveyed across the Indo‐Pacific associate with this kind of endosymbiont (LaJeunesse et al., 2010). Increasing scrutiny of the ‘C15’ symbionts in colonies of *Porites* representing different species and geographic regions has revealed some genetic variation suggesting ecologically different symbiont ‘species’ (Camp et al., 2019; LaJeunesse, 2005; LaJeunesse et al., 2010; Terraneo et al., 2019). Given the increasing importance of *Porites* species to the maintenance of reef growth and ecosystem function in warming seas, a basic understanding of how physiological variability among them may be influenced by harboring different, albeit closely related, symbionts is critical toward understanding patterns of bleaching and mortality within and between *Porites* species.

Little is known about the extent to which endosymbionts regulate the degree of thermal tolerance among *Porites*. Given the broad range of reef environments in Palau, this location provides an ideal system to examine whether genetic differences among *Cladocopium* C15 also correspond to important physiological differences among *Porites* spp. Here we characterized the symbiont genotypes and phenotypes in populations of two common Indo‐Pacific corals, *Porites rus* and *Porites cylindrica*, collected from inshore and offshore reef habitats. Reef corals receive significant metabolic resources from their symbiotic dinoflagellates (Ferrier‐Pagès et al., 2021; Hoadley et al., 2016; Muscatine, 1990); hence, algal symbiont physiological characteristics, such as light harvesting, carbon production, and nutrient translocation are key functional traits that, along with host physiology (Baird et al., 2009; Hume et al., 2013), help explain phenotypic variability across many species of reef‐building coral (Suggett et al., 2017). A short‐term heating experiment was therefore employed to examine differences between these different *Porites* species and how their habitat of origin and resident symbiont influenced their stress responses by examining photochemistry, carbon fixation, and the fate of photochemically fixed carbon toward coral calcification. A rapidly evolving genetic marker was used to interpret whether observed differences in physiology were due in part to the existence of previously unrecognized diversity within the *Cladocopium C15* group.

## 2 MATERIALS AND METHODS

### 2.1 Coral collection

Experiments were conducted in two consecutive years. *Porites rus* was tested in March 2014 and *Porites cylindrica* was tested in March 2015. In each year, offshore corals were collected from Rebotel reef (7°14.93’N, 134°14.149’E) and inshore corals were collected in Nikko Bay (7°32.48’N, 134° 49.34’E). Eight colonies species^−1^ site^−1^ were collected at a depth of 5 m (for Nikko Bay) and 10 m (Rebotel reef). At these respective depths, PAR measurements indicated irradiance levels to be roughly 800 μmol quanta m^−2^ s^−1^ at both sites. All sampled colonies were free of any visible signs of disease or physical damage and colonies were collected at least 10 m apart to better ensure genetic variability across colonies. Corals were transported to the Palau International Coral Research Center (PICRC) and fragmented into seven replicate nubbins and placed into a large (1200 l) outdoor flow‐through tank exposed to natural sunlight (peak intensity 800 μmol quanta m^−2^ s^−1^ with neutral density screening) with high water movement provided by several submersible powerhead pumps. The tank water temperature was set to 27.5℃ (which reflects the mean monthly average of Palau in March—27.8℃; NOAA coral watch) and controlled by a submersible chilling coil. Satellite‐derived sea‐surface temperature profiles (NOAA—coral reef watch) suggest similar thermal histories for reef corals preceding and during experiments in each year (Figure S1 and Table S1 for monthly means, standard deviations, and statistical comparison across years). Seawater was pumped from the PICRC pier at a depth of 3 m and then passed through a pressurized sand filter and aquarium sediment filters prior to use in flow‐through holding and experimental systems. One day after fragmentation, coral nubbins were attached to 2‐inch square PVC tiles with marine epoxy (Splash zone compound A‐788). Corals were then held at ambient conditions as described above for 12–16 days prior to starting each experiment to allow time for acclimation to the holding light conditions (Anthony & Hoegh‐Guldberg, 2003).

### 2.2 Experimental System

Each treatment system consisted of between 7 and 12 (56 L) plastic treatment bins held outdoors and fed by a central (~1200 L) head tank. Temperature conditions within the head tanks were maintained via a chiller system and aquaculture titanium heaters. All experimental bins were placed underneath an overhead canopy covered by a clear plastic (non‐UV filtering) sheet to block periodic rainfall and a 60% shade cloth allowing for a peak midday light intensity of 800 μmol quanta m^−2^ s^−1^. Light data measured with a spherical quantum PAR sensor and data logger (LI‐COR) beneath the shade canopy for each treatment year are provided in Figure S2. For the high temperature treatments, the temperature was gradually ramped from 27.5 to 32℃ over 4 days, and then held at 32℃ (31.71 ± 0.27℃ and 31.86 ± 0.47℃ for years 2014 and 2015, respectively, as recorded using HOBO Pro v2 underwater temperature loggers; Onset Computer Corporation, Bourne, MA, USA; sampled every hr; resolution ±0.2C) for an additional 10 days (14 days, total heating). For Palau, the maximum sea surface temperatures for inner and outer reefs are 31.2 and 30.7℃, respectively (Woesik et al., 2012). Because our corals reflect both inshore and offshore sites, a high temperature setting of 32℃ reflects a middle ground of roughly 0.8–1.3℃ above the average maximums expected. Temperature within the ambient control treatments was maintained at 27.5℃ throughout the experiment (27.44 ± 0.07℃ and 27.61 ± 0.26℃ for years 2014 and 2015, respectively). All bins and PVC tiles were regularly cleaned to prevent algal fouling. For each treatment, fragments from each colony were placed within separate bins and moved between and within bins daily so as to reduce possible tank effects.

### 2.3 Non‐destructive measurements and symbiont photophysiology

The dark acclimated maximum quantum yield of photosystem II (Fv/Fm^MT^) was measured every other day, 1 h after sunset by pulse amplitude modulation fluorometry (Diving PAM, Walz, Germany). Fluorescence was measured in three separate locations using a 0.6‐second multiple turnover saturation pulse (saturation intensity >4000‐μmol quanta m^−2^ s^−1^). Fv/Fm^MT^ values were then averaged for each fragment for a colony mean Fv/Fm^MT^.

On day 14, photosynthesis and respiration rates were measured from all fragments. Maximum net photosynthesis (P_max_) was measured by oxygen evolution with Fibox 4‐fiber optic O_2_ sensors (PreSens) housed in custom clear acrylic chambers (300 ml). Chambers were held in a water bath set to the control or experimental temperature. Filtered (0.45 μm) natural seawater in each chamber was continually circulated by a magnetic stir bar, and chambers were illuminated from above at 500 μmol quanta m^−2^ s^−1^ by a custom 24‐LED array (Cree Cool White XP‐G R5). Zero change in oxygen concentrations was observed for a blank (no coral) chamber containing only filtered seawater. Pilot studies confirmed that both corals from each location reached maximum photosynthesis (P_max_) at this light level. Net oxygen production was recorded for 15 minutes in the light (P_net_), followed by a 15‐minute dark interval to record the light‐acclimated dark respiration (R_L_). Gross photosynthesis (P_G_) was calculated as P_G_ = P_net_ − R_L_ and normalized to algal cell number.

Active chlorophyll *a* fluorescence was also measured at the end of the heating experiment on day 14 with a Fluorescence Induction and Relaxation (FIRe) fluorometer (Satlantic Inc., Halifax) fitted with a fiber optic LED excitation light source (peak λ 455 nm) (Hennige et al., 2011). After dark acclimating for 30 min, fluorescence measurements consisted of five iterations of a 100 *μ*s single turnover flash followed by a 2000 *μ*s relaxation phase consisting of 1 *μs* light flashes spaced 59 *μs* apart. The single turnover flash was followed by a 100 ms multi‐turnover flash and relaxation phase. FIRe data were used to calculate the maximum PSII quantum yield by a single turnover flash (Fv/Fm^ST^), PSII functional absorption cross‐section (σ_PSII_), the time constant for photosynthetic electron transport on the acceptor side of PSII (τ_PSII_), and the time constant for plastoquinone pool reoxidation (τ_PQ_).

Photosystem II electron transport rate (ETR) and non‐photochemical quenching of chlorophyll *a* fluorescence (NPQ) were measured after incubating coral fragments under 500 μmol quanta m^−2^ s^−1^ white light (RG5‐cool white CREE LED) for 5 minutes. ETR per reaction center was calculated as:

$${\text{ETR}}^{\text{RCII}}=\text{PFD}\times F_{q}\text{’}/F_{m}^{\text{’}\text{ST}}\times \sigma_{\text{PSII}}\times \mathrm{21.683}$$

where PFD is the photon flux density, F_q_^’^/F_m_’^ST^ is the operating efficiency of PSII in the light‐acclimated state, σ_PSII_ is the functional absorption cross‐section of PSII in the dark, and 21.683 converts seconds to hours, *µ*mol e^–^ to mol e^–^ and Å^2^ quanta^−1^ to m^2^ mol RCII^−1^ (Suggett et al., 2003). NPQ was calculated as:

$$\text{NPQ}=\left({\text{Fm}}^{\text{MT}}–{\text{Fm}}^{\text{MT}}\text{’}\right)/{\text{Fm}}^{\text{MT}}\text{’}$$

where Fm is the maximum dark‐acclimated fluorescence and Fm’ is the light acclimated maximum fluorescence, both recorded after the 100 ms saturation pulse. All fluorescence parameters listed above were calculated by fitting each fluorescence curve with *FIREPRO* software (Kolber & Falkowski, 1998).

### 2.4 Stable isotope pulse labeling

On day 14, control and treatment fragments were placed into glass beakers containing 400 mL of freshly filtered sea water (0.45 μm) that was enriched with 0.633 mM of NaH^13^CO_3_ (99 atom % ^13^C, Cambridge Isotope Lab Inc., Andover, MA, USA), giving a final 38% ^13^C seawater enrichment. Beakers were fitted with magnetic stir bars with false bottoms, continually stirred, and held in the same system described above for oxygen measurements, under the same constant light level, for 5 h. Preliminary measurements determined that this H^13^CO_3_ concentration was not limiting for this incubation time. Fragments were then removed, rinsed in filtered seawater, and immediately processed as described below for the algal and coral skeletal samples.

### 2.5 Sample processing for host and symbiont physiological metrics

Host and symbiont physiology were measured from treatment and control fragments on day 14. Coral tissue was removed by airbrush (100 psi) with filtered seawater (0.45 μm) (Szmant & Gassman, 1990). The resulting slurry was homogenized with a Tissue‐Tearor (Biospec products, Inc), and then divided into 1 or 2 ml aliquots. One 1 mL aliquot was preserved with 0.5% glutaraldehyde and stored at 4℃ for counting algal cells while another aliquot was resuspended in a DNA preservation buffer (Seutin et al., 1991) and stored at 4℃. All 2 ml aliquots were centrifuged for 2 min (5,000 *g*) and the supernatant was discarded. The resulting algal pellets were immediately frozen (−20℃) shipped back to the United States, and stored at −20℃ for further processing (chlorophyll, soluble protein, carbohydrates, and lipids). Coral fragment surface area was determined by the hot wax method (Stimson & Kinzie, 1991).

### 2.6 Symbiont identification

Symbionts were genetically characterized using rDNA and chloroplast gene markers. DNA extracts were performed as described in Hoadley et al., (2019). The internal transcribed spacer 2 region (ITS2) of the ribosomal array was amplified, followed by denaturing gradient gel electrophoresis (DGGE) to evaluate the presence of intragenomic rDNA variation and co‐occurring symbionts. Products from these PCRs were electrophoresed on denaturing gradient gels (45%–80%) using a CBScientific system (Del Mar, California, USA). Bands diagnostic of repeated fingerprints (i.e., diagnostic of a particular symbiont type) were cut from the gel, re‐amplified, and cycle sequenced (LaJeunesse, 2002; LaJeunesse et al., 2003, 2010; Sampayo et al., 2009). The psbA non‐coding region was also amplified using the primers: 7.4forw (GCA TGA AAG AAA TGC ACA CAA CTT CCC) and 7.8rev (GGT TCT CTT ATT CCA TCA ATA TCT ACT G) using the PCR conditions optimized by Moore et al., (2003). Using the 7.4forw primer, the 5‐prime end of *psbA* ^ncr^ (~400 bases) was directly Sanger sequenced using Big Dye 3.1 reagents (Thermo Fisher Scientific, Waltham, Massachusetts, USA) and the reactions were analyzed on an Applied Biosystems 3730XL instrument at the Penn State genomics core facility. Full sequences of the PsbAncr were deposited in Genebank (Accession numbers AA000000–AA000000). Phylogenetic analyses using maximum parsimony and maximum likelihood were conducted on aligned sequences using PAUP; v4.0a136 (PAUP, 2003). Bootstrap support of branching nodes was based on 1000 replicates.

### 2.7 Symbiont number and volume

Algal cells were counted with a hemocytometer (n = 4 counts sample^−1^) and photographed using an epi‐fluorescent microscope (EVOS, 40× magnification) and analyzed using the software Image J (NIH) with the Analyze Particles function to calculate algal cell volume as described in Hoadley et al., (2019).

### 2.8 Algal biochemical composition and photopigment concentration

For each assay, two technical replicates were run sample^−1^ replicate, and absorbance was determined with a FLUOstar Omega plate reader (BMG Labtech, Germany). For soluble algal protein and carbohydrates, cells were lysed in 2 ml of filtered seawater in a bead‐beater (BioSpec) for 2 min. The protein concentration was determined from 50 μL subsamples with the BCA colorimetric assay (λ 595 nm) (Thermo Scientific Pierce) with a bovine serum albumin standard (Smith et al., 1985). Carbohydrates were quantified from a 100 μL subsample by the sulfuric acid/phenol method (λ 485 nm) with a glucose standard (Dubois et al., 1956). For lipids, the algal pellet was freeze‐dried overnight and then extracted in a chloroform:methanol:sodium chloride mixture (2:1:0.8) (Folch et al., 1957). Lipids were quantified by a sulfo‐phospho‐vanillin colorimetric assay (λ 540 nm) with a corn oil standard (Cheng et al., 2011). Chlorophyll *a* was extracted by resuspending the algal pellet in 90% methanol and bead beating for 2 min. Samples were incubated at −20℃ for 2 h, and then centrifuged (2300 g) for 5 min. The resulting supernatant was measured at λ 665, 652, and 750 nm and chlorophyll *a* calculated by published equations (Porra et al., 1989). Protein, lipid, carbohydrate, and chlorophyll *a* data were normalized to algal cell number.

### 2.9 Stable isotope analyses

Coral tissue was removed with an air‐brush as previously described, followed by the addition of 0.02% (w/v) sodium dodecyl sulfate (SDS) and homogenization for 10 s with a Tissue‐Tearor (Biospec products, Inc). Symbiotic algae and coral tissue were separated by 2–3 centrifugation washes (550 *g* for 5 min) with 10 s homogenization between each wash (Lesser & Shick, 1989). Algal fractions were microscopically verified to ensure homogeneity and removal of the bulk of the animal material. Clean algal cells were pelleted via centrifugation (5,000 *g* for 5 min) and frozen at −20℃ until analyzed. Due to the relatively high concentration of ^13^C assimilation by the symbiotic algae during the labeling incubations, coral skeletons were placed in 100% bleach for 24h to remove remnant organic material from host–algal tissue, and then rinsed in fresh water for 24h, and dried under low heat. Approximately 20 mg of the outermost CaCO_3_ was sampled from both the corallite and coenosarc regions of the coral skeleton using a Dremel tool with a diamond bit. Skeletal samples were stored at −20℃ until analyzed. Elemental ^13^C analyses were performed on a Carlo Erba CHN Elemental Analyzer (Model NA1500) coupled to Thermo Finnigan Delta V Isotope Ratio Mass Spectrometer via a Thermo Finnigan Conflo III Interface at the University of Georgia, Center for Applied Isotope Studies. Isotopic data are expressed in delta (δ) notation in units per mil (‰) (Fry, 2006).

### 2.10 Statistical Analyses

**FIGURE 1:**
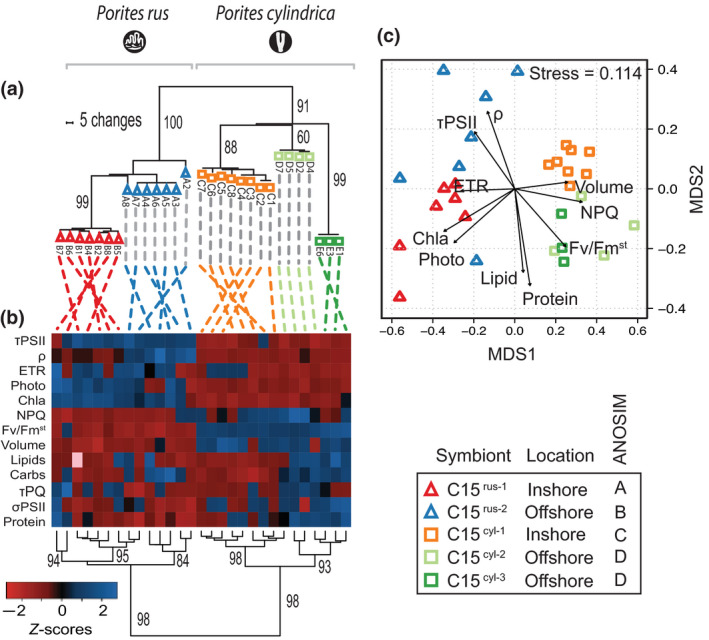
Genotype to phenotype analysis of *Porites* photosynthetic symbionts: Panel (a) represents a phylogenetic reconstruction based on the non‐coding region of the *psb*A minicircle depicting symbiont identities among *P*. *rus* (triangles) and *P*. *cylindrica* (squares) coral species. Bootstrap values based on 1000 iterations are indicated below branch lengths. The heat map in panel (b) reflects symbiont‐centric physiological metrics displayed in standard deviation from the mean (Z‐scores) and are taken from control (27.5℃) samples on day 14. Average values can be found in Supplemental Table 2. Sample order was determined through hierarchical clustering reflected throughout the heat map dendrogram. Bootstrap values are based on 1000 iterations and are indicated for major nodes. Multidimensional scaling of symbiont variables is reflected in panel (c). Physiological parameters are represented through vectors with significant (*p* < 0.001, 999 permutations) correlation to the plotted sample points

All analyses were conducted in R (v.3.5.1) (Team R. C., 2017). All 13 physiological variables in the control fragments were compared across each coral species and site by multivariate analysis of similarities (ANOSIM) with 9999 permutations in the vegan package (Oksanen et al., 2017). These variables included *F*_v_/*F*_m_^ST^, PSII connectivity (ρ), functional absorption cross‐section of PSII (σ_PSII_), the time constant for photosynthetic electron transport on the acceptor side of PSII (τ_PSII_), the time constant for plastoquinone pool reoxidation (τ_PQ_), non‐photochemical quenching of chlorophyll *a* fluorescence (NPQ), electron transport rate (ETR), gross photosynthesis, lipid, carbohydrate, protein, cell volume, and chlorophyll *a* (Table 1). Because the range for each variable can differ considerably, individual measurements were normalized to their maximum value. These variables were also visualized in two‐dimensional space by non‐metric multidimensional scaling (nMDS) (Hoadley et al., 2019). Significant parameters (*p *< 0.001, 999 permutations) in the nMDS ordination are displayed as vectors in Figure 1c. Both vectors and nMDS plots were created using the vegan R package (Oksanen et al., 2017).

**TABLE 1:** Table of terms—definitions and units

Term	Definition	Units
τ_PSII_	Rate constant for reoxidation of the Q^a^ site of the D1 protein within the PSII RC	μ‐seconds
τ_PQ_	Rate constant for reoxidation of the plastoquinole pool.	μ‐seconds
ETR	RCII‐specific electron transport rate	mol e^−^ mol RCII^−1^h^−1^
NPQ	Non‐photochemical quenching	Relative units
*F*_v_/*F*_m_^ST^	Dark acclimated maximum quantum yield of PSII (single turnover)	Relative units
ρ	Connectivity between PSII reaction centers	Relative units
σ_PSII_	Dark acclimated effective absorption cross‐section of PSII	Å^2^q^−1^
Lipids	Lipid concentration cell^−1^	μg cell^−1^
Protein	Protein concentration cell^−1^	μg cell^−1^
Carbohydrates	Carbohydrate concentration cell^−1^	μg cell^−1^
Volume	Symbiont cellular volume	μm^−3^
Photo	Gross algal photosynthesis	mg O_2_ L^−1^ min^−1^ cell^−1^
Chla	Chlorophyll concentration cell^−1^	pg cell^−1^
*F*_v_/*F*_m_^MT^	Dark acclimated maximum quantum yield of PSII (multi‐turnover)	relative units
δ^13^C	Carbon isotope used for tracer	δ^13^C per mil (‰)

To compare individual coral fragments to their respective symbiont physiology, all ambient physiological variables for each fragment and site were also compared by an unsupervised hierarchical cluster analysis in the pvclust package (Suzuki & Shimodaira, 2013). Data were converted into z‐scores (mean‐sample and standard deviation) and 1000 bootstrap iterations were used to build a heat map and dendrogram.

The temperature response for all 13 algal physiological variables for each coral species from each location after 14 days of heating was analyzed by a one‐way multivariate analysis of similarities (ANOSIM) with 9999 permutations. Analyses of each individual variable between control and high temperature treatments were individually tested by a t‐test. The thermal response of the maximum quantum yield of PSII recorded through time by PAM fluorometry (Fv/Fm^MT^) was analyzed by a linear mixed model analysis for each coral species, with the coral colony set as a random factor in the lmerTest package (Kuznetsova et al., 2017). Isotopic (δ^13^C ‰) comparisons between control vs. heated algal and skeletal samples within each collection site/coral–algal group were analyzed by a paired t‐test.

For all univariate analyses (comparisons across coral species and reef sites or treatment conditions), datasets were initially tested for normality by a Shapiro–Wilks test. Non‐normal data were square‐root or log transformed and retested. For each physiological parameter, differences across coral species and reef sites were also assessed by a one‐way ANOVA with a Tukey post‐hoc test.

## 3 RESULTS

### 3.1 Symbiont genotype and population structure

Regardless of reef location (Offshore vs. Nikko Bay), analysis of the ITS2 rDNA region identified *Cladocopium C15* as the dominant symbiont in both *P*. *cylindrica* and *P*. *rus*. However, phylogenetic analysis of the psbA non‐coding region revealed five divergent taxonomic clusters that correspond to (1) separate coral species and (2) collection sites characterized by distinct environmental conditions (Figure 1a). Specifically, one unique *Cladocopium C15* genotype was noted in *P*. *rus* at each reef location (abbreviated hereafter as C15^rus−1^ and C15^rus−2^ for inshore and offshore locations, respectively). For *P*. *cylindrica*, a single unique *Cladocopium C15* genotype (C15^cyl−1^) was found inshore while two different genotypes were found among the offshore colonies, with four colonies each harboring genotype C15^cyl−2^ or C15^cyl−3^. All corals maintained algal specificity between ambient and elevated temperature treatments with the same symbionts as at the start of the experiment.

### 3.2 Baseline physiological differences across symbiont genotypes

When all 13 physiological metrics were compared in control coral fragments on day 14, there was significant separation across the *C15* genotypes (pairwise ANOSIM). Genotypes C15^rus−1^, C15^rus−2^, and C15^cyl−1^ were each distinct from all others while the two offshore genotypes within *P*. *cylindrica*, C15^cyl−2^ and C15^cyl−3^, significantly separated from all other algae but not each other (Figure 1, Table S2, S3, post‐hoc analyses for all 13 variables are provided in Table 2). Vectors within the nMDS ordination are maximally correlated to the plotted points and provide insight into which physiological metrics were most important for differentiating algal genotypes (Figure 1c). As an example, the volume and NPQ arrows suggest higher values for symbionts in *P*. *cylindrica*, whereas ETR is greater for symbionts in *P*. *rus*. The heat map in Figure 1b provides a detailed view of all physiological variables across each coral colony and a direct algal genotype to phenotype comparison. The heat map and corresponding cluster analysis (Figure 1b) strongly supported significant physiological differences between the algal populations within the two coral species. In addition, with the exception of a single C15^cyl−2^ sample, high cluster bootstrap values indicated that the C15^cyl−1^ genotype from Nikko Bay was phenotypically distinct from all other C15 symbionts (Figure 1b).

**TABLE 2:** Physiological differences across symbiont types. Significant results from one‐way ANOVA or Kruskal–Wallace are in **bold**. For each physiological metric, letters represent significant differences across genotypes based on post‐hoc analysis

Value	*p*‐value	Post‐hoc Results				
		C15^rus−1^	C15^rus−2^	C15^cyl−1^	C15^cyl−2^	C15^cyl−3^
τPQ	**0.0028**	B	A	AB	B	AB
τPSII	**<0.0001**	A	A	B	B	B
ETR	**0.0108**	A	AB	AB	B	B
NPQ	**<0.0001**	A	A	B	B	AB
FvFm^st^	**<0.0001**	A	A	B	B	C
ρ	**0.0002**	B	A	C	BC	C
σ_PSII_	**0.0044**	A	A	AB	A	B
Lipids	**0.0261**	AB	AB	A	B	B
Protein	**0.0014**	B	A	B	B	B
Carbs	**0.0016**	AB	A	B	AC	AC
Volume	**<0.0001**	A	AC	B	AB	BC
Photo	**0.0037**	A	AB	B	B	B
Chla	**0.0002**	A	A	B	B	AB

### 3.3 Symbiont and coral response to elevated temperature

**FIGURE 2:**
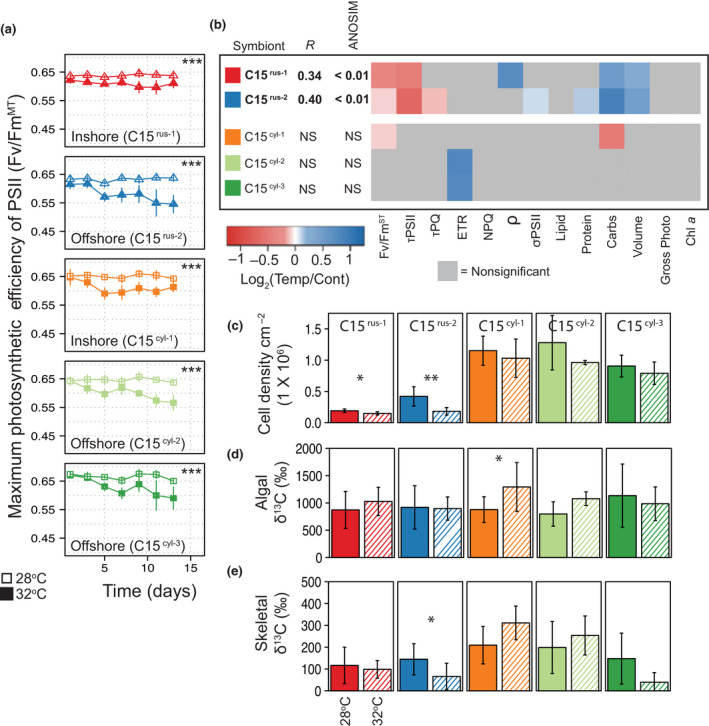
Physiological responses to elevated temperature: Maximum photosynthetic efficiency values (PAM based) were measured every other night throughout the 14‐day experiment and are plotted in panel (a). The heat map in panel (b) reflects the average Log^2^ fold change in response to elevated temperature for each physiological variable. Variables where no significant change in response to temperature was observed are displayed in gray. Bar graphs represent cell densities (c) and algal (d) and skeletal (e) δ^13^C (‰) under ambient and elevate temperature for each host/symbiont combination. Asterisks represent significant differences between control and high temperature treatments (*p < *0*.*05*, **p < *0*.*01*, ***p < *0*.*0001*). Errors bars reflect ±SD

Regardless of coral species or reef location, the maximum quantum yield of PSII (Fv/Fm^MT^, measured by PAM fluorometry) declined significantly over time in all heated corals relative to their respective controls (*p *< 0.0001) (Figure 2a). However, this loss in photochemical capacity was small, ranging from 2% to 11% and was always greater in the offshore corals.

When all 13 physiological variables were compared, significant differences between control and heated samples were noted only in the algae within inshore and offshore *P*. *rus* fragments (ANOSIM, R = 0.3827, *p *= 0.0018 for C15^rus−1^ and R = 0.5228, *p *= 0.0008 for C15^rus−2^) (Figure 2b). Pairwise comparisons within each coral–algal genotype pairing revealed a significant decline in both Fv/Fm^ST^ and the reoxidation rate of the PSII reaction center (τ_PSII_) in *P*. *rus* fragments (*p *= 0.0425, 0.0038 and *p *= 0.0006, 0.0036 for C15^rus−1^ and C15^rus−2^, respectively) (Figure 2b) while the plastoquinone pool (τ_PQ_) also dropped significantly in the C15^rus−2^ alga in offshore *P*. *rus* fragments (*p* = 0.0028) (Figure 2b). Meanwhile, PSII reaction center connectivity (ρ), carbohydrates, and cell volume increased significantly in heated samples in C15^rus−1^ (*p *= 0.0036, 0.001, and 0.0001, respectively). For the C15^rus−2^ alga in offshore *P*. *rus* fragments, carbohydrates and cell volume also increased significantly, as well as soluble protein and the functional PSII absorption cross‐section (σ_PSII_) (*p *= 0.0305, 0.0044, 0.0110, and 0.0028, respectively) (Figure 2b).

While there was no overall change across all heated *P*. *cylindrica* fragments (ANOSIM *p* >0.05), a few physiological metrics did change and were separated by coral location. Here, Fv/Fm^ST^ and carbohydrate concentrations decreased in the inshore C15^cyl−1^ alga (*p *= 0.0179 and 0.0402, respectively) while the PSII electron transport rate significantly increased in all offshore corals harboring either the C15^cyl−2^ or C15^cyl−3^ alga (*p* = 0.0023 and 0.0108, respectively).

After 10 days of heating, *P*. *rus* fragments from both reef locations lost a significant number of algal cells (*p *= 0.0117 and 0.0023 for C15^rus−1^ and C15^rus−2^, respectively), and the decline was markedly greater in the offshore fragments (21% vs. 56% loss for C15^rus−1^ and C15^rus−2^, respectively). In contrast, there was no significant decline in symbionts (*p *> 0.05) in any heated *P*. *cylindrica* samples (Figure 2c). There was no significant change in algal ^13^C incorporation between control and heated inshore or offshore *P*. *rus* fragments; conversely, the C15^cyl−1^ alga in the heated inshore *P*. *cylindrica* assimilated significantly more ^13^C than the control samples (*p *= 0.032) (Figure 2d). Similar to *P*. *rus*, there was no change in control vs. heated ^13^C fixation in the offshore *P*. *cylindrica* samples. While heating did not affect the amount of ^13^C incorporated into the inshore *P*. *rus* skeletons, significantly less ^13^C was detected in the heated offshore *P*. *rus* skeletons compared to the control samples (*p *= 0.047) (Figure 2e). Conversely, skeletal ^13^C increased by 49% in the heated inshore *P*. *cylindrica* samples and was marginally significant (*p *= 0.0511). Heating had no effect on isotopic labeling in the offshore *P*. *cylindrica* harboring C15^cyl−2^ and while the skeletal ^13^C signal in the heated *P*. *cylindrica* harboring C15^cyl−3^ decreased on average over 70%, and this drop was not statistically significant (Figure 2e).

## 4 DISCUSSION

Without the appropriate resolution of ecologically separate and evolutionarily divergent symbiodiniacean lineages, differences in acclimation and stress tolerance will remain unrealized among many host‐specific symbionts. Here we found a breadth of phenotypes in response to thermal stress among closely related endosymbiotic dinoflagellates, and revealed how their differences affected thermal tolerance among colonies from a dominant and critically important group of host corals. Accurate ecological predictions and subsequent conservation and management decisions are thus reliant on our ability to properly describe functional diversity across a myriad of host–symbiont relationships.

### 4.1 Differences in physiology and ecology among related symbionts

*Cladocopium* corresponding to type *C15*, including related ITS2 sequence variants (*C15a*, *C15b*, etc.), remains a cryptic sub‐clade within this genus of physiologically and ecologically differentiated micro‐algae commonly associated with the coral genera *Montipora* and *Porites* (LaJeunesse et al., 2010). While many have noted high thermal tolerance among *Cladocopium C15* (Fabricius et al., 2004; Fisher et al., 2012; Fitt et al., 2009; Hoadley, Pettay, et al., 2015; Wang et al., 2012), phenotypic differences also exist across this lineage. For example, when only a single ‘type’ of symbiont was identified (i.e., C15), differential bleaching between inshore and offshore *P*. *lobata* was attributed to genetic differences among coral colonies in each population (Barshis et al., 2010). Similarly, differences in susceptibility to thermal stress have been attributed to physiological differences between species or populations of *Porites* (Boulay et al., 2014; McClanahan, 2004). This current study is the first to document how closely related lineages within the *Cladocopium C15* group are physiologically distinct and influence how different *Porites* species and populations within species from different habitats cope with thermal stress.

Our discovery of five closely related, albeit divergent *Cladocopium* lineages associated with two species of *Porites* has significant implications for the functional ecology of each coral species living under different environmental conditions. Cluster analysis of 13 physiological proxies differentiates symbionts specific to each host species and reef environment. Trait‐based differences were largely concordant with the phylogenetic identity of the symbiont, which appears adapted to certain reef environments and particular host species (Figure 1a,b). Prior studies have also identified physiological differences across certain closely related symbionts. For example, *Breviolum sp*. isolated from various host cnidarians and experimentally manipulated in culture (Bayliss et al., 2019; Díaz‐Almeyda et al., 2017; Suggett et al., 2015) or in symbioses with the model system, *Exaiptasia pallida* (Hoadley et al., 2015; Leal et al., 2015). Differences in symbiont cell size and shape were congruent with genetic distinctions and ultimately lead to the recent classification of three new species of closely related *Breviolum* from Caribbean reef corals (Lewis et al., 2019). For members of *Cladocopium*, high levels of species diversity contribute to the differences in thermal tolerance and light acclimation noted across individual species (Abrego et al., 2008; Fitt et al., 2009; Hume et al., 2013; Rodriguez‐Lanetty et al., 2004). Phenotypic distinctions observed here and in prior studies will likely dictate the response of each mutualism to environmental change. Thus, community shifts in the diversity and abundance of reef‐building corals under continued ocean warming may depend largely on the unique characteristics of existing host–symbiont partnerships, even among closely related species of both coral and symbiont.

While thermal experiments on *P*. rus were conducted in 2014 and *P*. *cylindrica* in 2015, thermal histories and irradiance levels (Figures S1, S2 and Table S1) indicate similar conditions and allow for some comparison across host species, despite some environmental variability between years. The *Cladocopium* spp. associated with *P*. *rus* appeared more sensitive to thermal stress as more physiological metrics were altered under high temperatures than those harbored by *P*. *cylindrica* (Figure 2b‐c). Both multi‐ and single‐PSII (Fv/Fm^MT^ and Fv/Fm^ST^, respectively) turnover chlorophyll *a* fluorescence for C15^rus−1^ and C15^rus−2^ declined in heat‐treated colonies of *P*. *rus* originating from inshore and offshore habitats, respectively. Further analyses of Fv/Fm^MT^ and Fv/Fm^ST^ identified whether there was a disruption of the PSII reaction center and/or a subsequent interruption in electron transport through the plastoquinone (PQ) pool (Vredenberg et al., 2012). As assessed by a decrease in τ_PSII_, the electron turnover rate was relatively faster through the PSII reaction center for both C15^rus−1^ and C15^rus−2^ during high heat treatment (Figure 2b). Moreover, the decrease in τ_PQ_ for C15^rus−1^ indicated that electron transport through the plastoquinone pool was uninhibited by high temperature in both C15^rus−1^ and C15^rus−2^. This increased electron transport through the PSII reaction center or the PQ pool should facilitate higher quantum yields, because captured photons are more efficiently funneled through the photosynthetic apparatus. However, despite an increase in electron transport, the decrease in maximum PSII quantum yield indicated an energetic decoupling across the thylakoid membrane in *P*. *rus* symbionts (Tchernov et al., 2004). Energetic decoupling within the photochemical apparatus may have ultimately led to their apparent sensitivity to thermal stress and symbiont loss from *P*. *rus* corals.

In contrast to *P*. *rus* symbionts, symbiont cell numbers in *P*. *cylindrica* corals remained stable under high temperature. In addition, Fv/Fm^MT^ still declined across all three *P*. *cylindrica* symbionts but Fv/Fm^ST^ only declined in the inshore colonies. Because this loss in PSII activity did not coincide with losses in cell density, the decreased Fv/Fm^MT^ and Fv/Fm^ST^ likely signifies acclimation to high temperatures rather than a proxy of thermal stress. Increased electron transport rates in both offshore symbionts may also be indicative of acclimation to heating and suggests that these symbioses are particularly well suited to withstand high temperature conditions.

**FIGURE 3:**
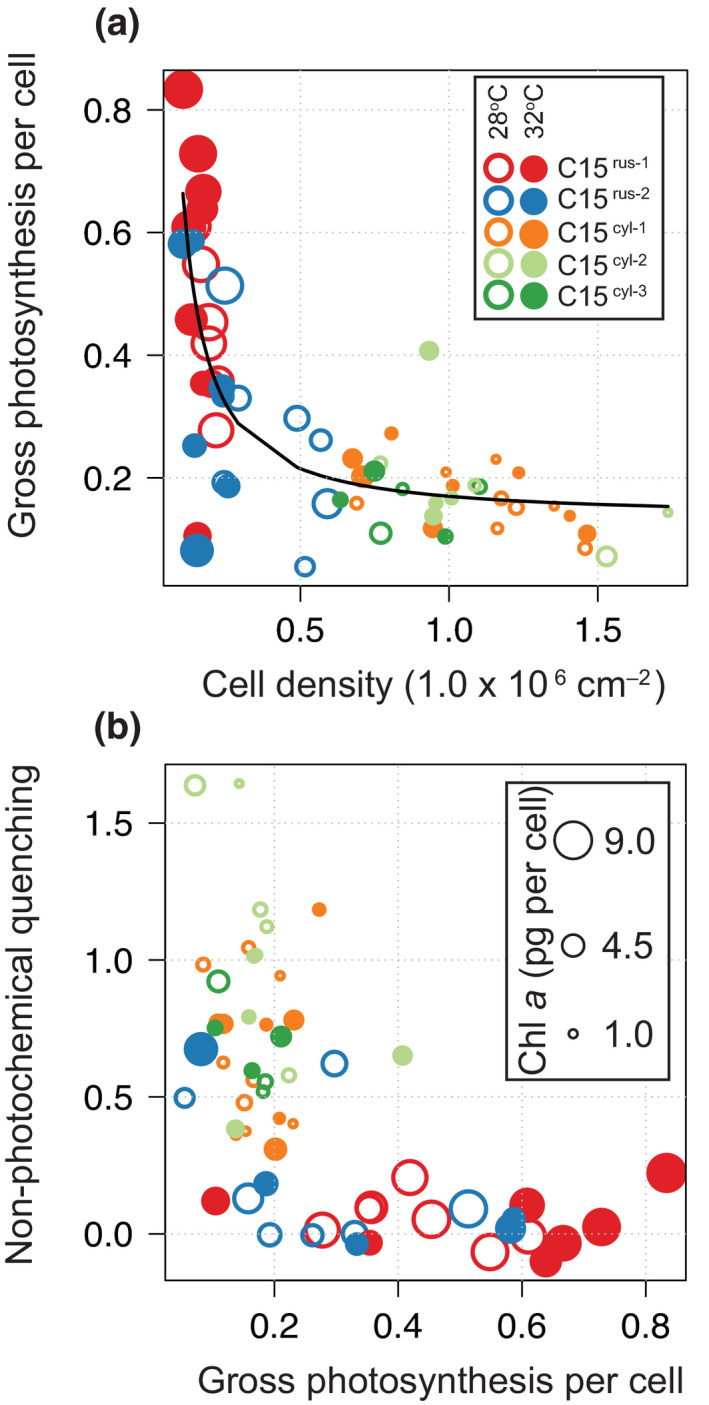
Correlation across algal physiological measurements: The top plot (a) reflects gross photosynthesis cell^−1^ (y‐axis) and cell density cm^−2^ (x‐axis). The bottom plot (b) reflects non‐photochemical quenching (y‐axis) and gross photosynthesis cell^−1^ (x‐axis). Symbiont identity is indicated by color (open circles = ambient, closed circles = elevated temperature). Shape size reflects Chl *a* concentration

A common photoprotective pathway among many photosynthetic organisms is to dissipate potentially damaging excess light energy as heat, which is often quantified as the non‐photochemical quenching (NPQ) of chlorophyll *a* fluorescence (Buck et al., 2019; Masojidek et al., 1999; Olaizola et al., 1994). Similarly, NPQ mechanisms are employed by symbiotic algae to mitigate light stress and/or photo‐damage during high temperature events (Hennige et al., 2008; Hill et al., 2004; Middlebrook et al., 2008). At ambient temperatures, symbionts within *P*. *rus* had significantly lower NPQ when compared to those harbored by *P*. *cylindrica* (with the exception of C15^cyl−3^) and may suggest greater tolerance to high light in C15^rus−1^ and C15^rus−2^. Both the inshore and offshore symbionts of *P rus* contained significantly more chlorophyll *a* cell^−1^, potentially increasing self‐shading and minimizing the need for elevated NPQ. (Figure 3b and Table 2). In addition, energetic connectivity among PSII reaction centers (ρ) was significantly higher and PSII reaction center reoxidation (τ_PSII_) was significantly faster in C15^rus−2^ as compared to C15^rus−1^ (Figure 1b, Table 2). Higher connectivity between PSII reaction centers enhances the probability that energy absorbed by chlorophyll surrounding a closed PSII reaction center will be transferred to a nearby open reaction center (Kolber & Falkowski, 1998; Xu et al., 2017). Additionally, faster PSII re‐oxidation (τ_PSII_) increases the rate of electron transport through the photosynthetic apparatus (Kolber & Falkowski, 1998). Taken together, these photochemical processes, combined with the low NPQ, higher cellular photosynthesis, and chlorophyll *a* cell^−1^, help to explain why the symbionts in *P*. *rus* may be better at light harvesting and photosynthesis under normal conditions, but are more sensitive to physiological stress when exposed to high temperature.

### 4.2 The effect of host‐symbiont combinations on the performance of the mutualism under stress

Photosynthetically fixed carbon translocated from the algal symbiont promotes calcification by the host coral (Colombo‐Pallotta et al., 2010; Furla et al., 2000; Goreau, 1959; Muscatine, 1990). High temperature stress to photosynthesis should therefore reduce skeletal growth. However, for *P*. *rus*, the decrease in Fv/Fm^MT^ and symbiont cell densities in heated corals did not lead to subsequent changes in gross photosynthesis per cell relative to controls (Figure 2b). Likewise, isotopic ^13^C incorporation was no different between heated and control samples for *P*. *rus* or *P*. *cylindrica*. A notable exception was in C15^cyl−1^ from inshore *P*. *cylindrica*, where carbon assimilation increased with heating (Figure 2d). Although photosynthetic rates (estimated using oxygen evolution) were maintained in C15^rus−2^ in heated offshore *P*. *rus*, substantial reductions of ^13^C incorporation into the coral skeleton indicate a redistribution of metabolic carbon within the symbiont and/or coral host, or a disruption in the biochemical processes of calcification (Figure 2e). Future work with more refined pulse‐chase isotopic methods (Loussert‐Fonta et al., 2020; Sproles et al., 2020) would resolve the relative importance of these possibilities. Specific host–symbiont combinations can therefore have significant consequences in colony performance (growth, health, stability of the mutualism) under different environmental contexts, even when involving closely related hosts and symbionts.

Our results challenge the general notion that corals with higher symbiont cell densities are more sensitive to thermal stress (Cunning & Baker, 2013). Increased reactive oxygen species (ROS), including peroxides and super oxides, are thought to contribute to the breakdown of the mutualism by causing molecular and cellular damage (Hawkins & Davy, 2012; Hawkins et al., 2015; Krueger et al., 2015; Nielsen et al., 2018; Weis, 2008). It is often assumed that higher symbiont densities produce more ROS when heated, further exacerbating the bleaching response (Cunning & Baker, 2013, 2014; Wooldridge et al., 2017). However, colonies of *P*. *cylindrica* contained significantly greater symbionts cm^−2^ than *P*. *rus*, yet it well tolerated the heat stress. However, prior studies focused on intraspecific symbiont cell densities and the resulting effect on coral thermal tolerance may not apply to the interspecific symbiont comparisons here.

These experiments determined that different host species have innately different standing symbiont cell densities (Stimson et al., 2002); and that such differences are important factors in the physiological performance of the resident symbiont (see below). Overall, both C15^rus−1^ and C15^rus−2^ in *P*. *rus* had higher gross photosynthesis cell^−1^, compared to C15^cyl−1^, C15^cyl−2^, and C15^cyl−3^ within *P*. *cylindrica* (Figure 3a, Table 2). The inverse relationship between total symbiont density and gross photosynthesis algal cell^−1^ is in agreement with other studies that have attributed lower cell densities to greater availability of inorganic or organic carbon to support photosynthesis (Hoadley et al., 2016; Rädecker et al., 2017; Tansik et al., 2017). Despite the significant decline in symbiont cells and PSII photochemistry, greater carbon availability cell^−1^ may have also contributed to sustained gross photosynthesis and ^13^C incorporation in the heat‐treated *P*. *rus*. This agrees with other studies that documented an inverse relationship between symbiont cell densities and oxygen production during high temperature stress among different coral species (Hoadley et al., 2016; Middlebrook et al., 2010).

The different thermal responses across closely related *Cladocopium C15* in this study cautions against broad assumptions of thermal sensitivity based on preliminary genetic characterizations. Marked functional differences exist even among closely related symbionts that, when responding to environmentally stressful conditions, may affect the stability of specific mutualistic partnerships. A holistic approach of characterizing multiple physiological traits is therefore essential for forecasting how the membership of coral communities from different habitats, with various environmental gradients, will respond to increasing stress in the context of global climate change (Litchman & Klausmeier, 2008; Madin et al., 2016; Weithoff, 2003). Under future climate conditions, the genus *Porites* spp. will continue to be a critical reef‐building coral. However, subtle phenotypic differences across their closely related symbionts may substantially affect their diversity, abundance, and community stability in the coming decades.

## DATA AVAILABILITY STATEMENT

Data and R scripts for Figures 1, 2 and 3 are available via github (khoadley/Porites_2021).

## AUTHORS’ CONTRIBUTIONS

D.K., T.L., and M.W. planned and designed the research. K.H., A.L., D.W., D.P., R.S., D.K., T.L., and M.W. performed the experiments, conducted the fieldwork, and analyzed the data; K.H., C.G., D.W., and A.L. processed the field samples and performed the further analyses; K.H., D.K, T.L., and M.W. wrote the manuscript; K.H. agrees to serve as the author responsible for contact and ensures communication.

## Supporting information

Supplementary Material
