# Supplementary material for: Different functional traits among closely related algal symbionts dictate stress endurance for vital Indo‐Pacific reef‐building corals
Source: Glob Chang Biol. 2021 Aug 2;27(20):5295–309. doi: 10.1111/gcb.15799 (PMC9291761; doi:10.1111/gcb.15799)
Supplement: Supplementary file 1 — Supplementary Material [file GCB-27-5295-s001.pdf]

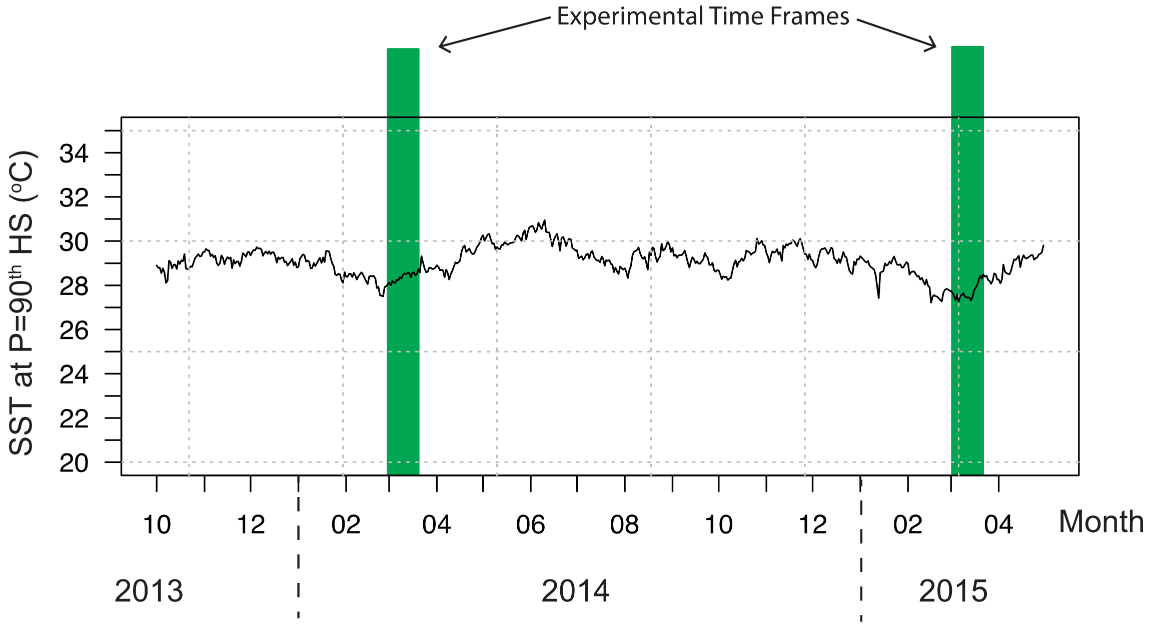

Supplemental Figure 1: Sea Surface temperatures derived from NOAA coral reef watch (CRW) virtual station for Palau (http://coralreefwatch.noaa.gov/satellite/vs/index.html). Temperatures during experimental time frames in 2014 and 2015 appear to vary less than 0.5 ˚C, with similar thermal histories over the previous few months (gradual decline in temperature from 30 to 27.5 ˚C).

Table S1: Analysis of temperature trend data, showing mean and standard deviation for each month. Granger test on the causality and predictive ability of each dataset on the other.

**Month**	**Year 2014 (mean ± sd)**	**Year 2015 (mean ± sd)**	**Mean Difference**
**October**	28.93 ± 0.32	29.12 ± 0.58	0.19
**November**	29.23 ± 0.23	29.61 ± 0.30	0.38
**December**	29.32 ± 0.26	29.21 ± 0.34	0.11
**January**	28.97 ± 0.37	28.86 ± 0.38	0.11
**February**	28.22 ± 0.32	27.99 ± 0.48	0.23
**Granger Test**	**model data**	**prediction**	**p-value**
	Oct ‘13 – Mar ‘14	Oct ‘14 – Mar ‘15	0.0169
	Oct ‘14 – Mar ‘15	Oct ‘13 – Mar ‘14	0.0094

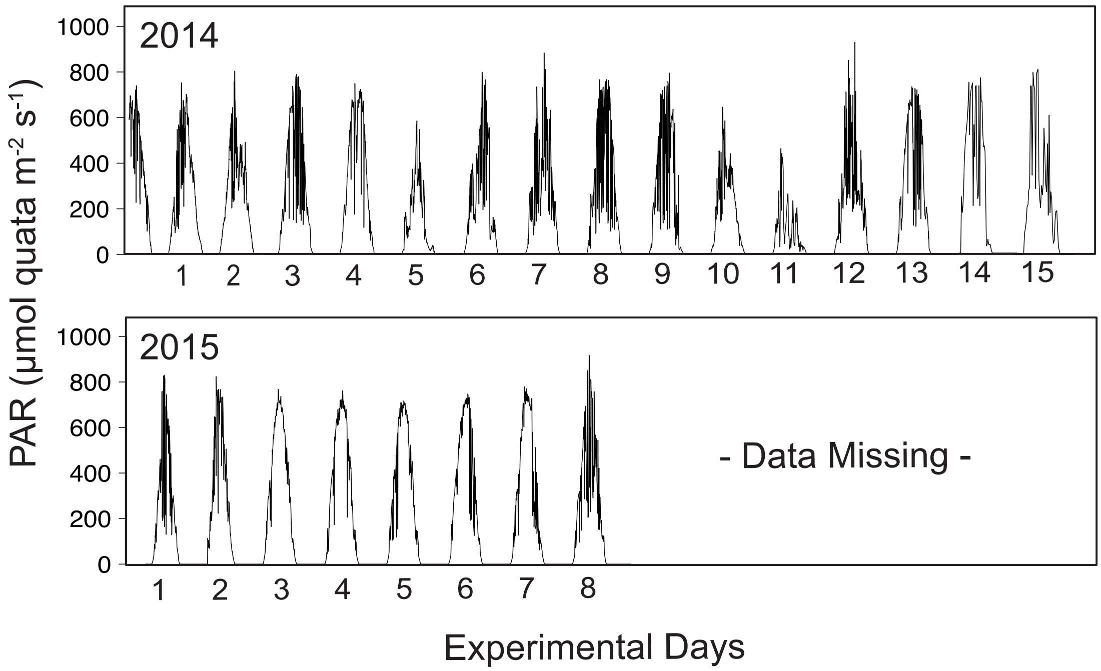

Supplemental Figure 2: Irradiance measurements over experimental days for each year. PAR readings were taken next to experimental tanks every 5 minutes using a 4pi LI-COR light meter. The last half of the data from 2015 is missing due to instrumentation errors.

Supplemental Table 2: Averages and SD for each symbiont type and physiological variable. Units for each metric are as described in table 1.

Value	Average ± SD				
	$C15^{rus-1}$	$C15^{rus-2}$	$C15^{cyl-1}$	$C15^{cyl-2}$	$C15^{cyl-3}$
τPQ	25234 ± 6980	18221 ± 2725	20547 ± 1609	23128 ± 1929	20307 ± 1086
τPSII	1889 ± 402	1942 ± 220	1297 ± 257	1133 ± 249	1185 ± 94
ETR	431879 ± 179563	356064 ± 167245	263213 ± 99178	175968 ± 50590	147322 ± 16573
NPQ	0.214 ± 0.163	0.355 ± 0.209	0.713 ± 0.212	1.229 ± 0.489	0.582 ± 0.176
Fv/Fm^{st}	0.447 ± 0.023	0.430 ± 0.014	0.504 ± 0.007	0.500 ± 0.014	0.556 ± 0.010
P	0.074 ± 0.008	0.163 ± 0.059	0.049 ± 0.008	0.053 ± 0.019	0.043 ± 0.006
Sigma	172.57 ± 13.16	163.57 ± 9.14	175.13 ± 6.94	170.75 ± 14.71	196.33 ± 10.79
Lipids	4.44E-04 ± 1.78E-04	4.28E-04 ± 2.52E-04	3.32E-04 ± 6.43E-05	6.68E-04 ± 1.58E-04	7.42E-04 ± 2.48E-04
Protein	9.10E-04 ± 3.36E-04	5.8E-04 ± 2.22E-04	9.30E-04 ±1.53E-04	12.0E-04 ±2.18E-04	12.5E-04 ± 2.60E-04
Carbs	8.60E-04 ± 2.11E-04	13.7E-04 ± 4.75E-04	7.3E-04 ± 2.15E-04	12.5E-04 ± 2.25E-04	12.9E-04 ± 1.51E-04
Volume	349.5 ± 43.5	373.4 ± 26.0	484.8 ± 59.2	435.3 ± 72.1	461.2 ± 36.4
Photo	4.31E-09 ± 1.17E-09	2.58E-09 ± 1.46E-09	1.59E-09 ± 4.62E-10	1.57E-09 ± 6.54E-10	1.59E-09 ± 4.27E-10
Chla	8.49 ± 1.17	5.92 ± 1.92	1.20 ± 0.82	1.88 ± 1.56	2.68 ± 1.59

Supplemental Table 3: ANalysis Of SIMilarity (ANOSIM with 9,999 permutations), for day 14. Comparison of ambient temperature symbiont physiology across C15 genotypes.

C15 genotype	R	*p*-value
**$C15^{rus-1}$ vs. $C15^{rus-2}$**	**0.412**	**0.0042**
**$C15^{rus-1}$ vs. $C15^{cyl-1}$**	**0.991**	**0.0002**
**$C15^{rus-1}$ vs. $C15^{cyl-2}$**	**1.000**	**0.0046**
**$C15^{rus-1}$ vs. $C15^{cyl-3}$**	**1.000**	**0.0126**
**$C15^{rus-2}$ vs. $C15^{cyl-1}$**	**0.945**	**0.0001**
**$C15^{rus-2}$ vs. $C15^{cyl-2}$**	**0.965**	**0.0025**
**$C15^{rus-2}$ vs. $C15^{cyl-3}$**	**0.948**	**0.0081**
**$C15^{cyl-1}$ vs. $C15^{cyl-2}$**	**0.674**	**0.0040**
**$C15^{cyl-1}$ vs. $C15^{cyl-3}$**	**0.814**	**0.0064**
$C15^{cyl-2}$ vs. $C15^{cyl-3}$	0.166	0.1714
